# Human Mercury Exposure in Yanomami Indigenous Villages from the Brazilian Amazon

**DOI:** 10.3390/ijerph15061051

**Published:** 2018-05-23

**Authors:** Claudia M. Vega, Jesem D.Y. Orellana, Marcos W. Oliveira, Sandra S. Hacon, Paulo C. Basta

**Affiliations:** 1 Center for Amazonian Scientific Innovation, Wake Forest University, 1834 Wake Forest Road P.O. Box 7306, Winston-Salem, NC 27106, USA; vegacm@wfu.edu; 2 Instituto Leônidas e Maria Deane, Fundação Oswaldo Cruz, Rua Teresina, 476, Adrianópolis, Manaus CEP: 69057-070, Brazil; jesem.orellana@gmail.com or jesem.orellana@fiocruz.br; 3 Instituto Socioambiental—ISA, Av. Higienópolis, 901, Higienópolis, São Paulo CEP: 01238-001, Brazil; marcos@socioambiental.org; 4 Escola Nacional de Saúde Pública Sérgio Arouca, Fundação Oswaldo Cruz, Rua Leopoldo Bulhões, 1480, Manguinhos, Rio de Janeiro CEP: 21041-210, Brazil; sandrahacon@gmail.com

**Keywords:** mercury exposure, indigenous, Brazilian Amazon, environmental, public health, epidemiology

## Abstract

In the Brazilian Amazon, where the majority of Yanomami villages are settled, mercury (Hg) exposure due to artisanal small-scale gold mining (ASGM) has been reported since the 1980s. This study assessed mercury exposure in the Yanomami reserve and whether the level of contamination was related to the ASGM geographical location. It was conducted using a cross-sectional study of 19 villages. Direct interviews were performed and hair samples were used as a bioindicator of Hg exposure. The Prevalence-Ratio (PR) was estimated as an indicator of association between ASGM geographical locations and human exposure to mercury. Mercury levels (239 hair samples) ranged between 0.4 and 22.1 μg·g^−1^ and presented substantial differences amongst the villages. In the Waikas-Aracaça region, where current ASGM was reported, we observed the highest Hg concentrations (median = 15.5 μg·g^−1^). Almost all participants presented with hair-Hg levels >6 μg·g^−1^ (prevalence = 92.3%). In the Paapiu region, we observed the lowest concentrations (median = 3.2 μg·g^−1^; prevalence = 6.7%). Our findings showed that the Waikas Ye’kuana and Waikas Aracaca villages presented with 4.4 (PR = 4.4; Confidence Interval (CI) 95% = 2.2–9.0) and 14.0 (PR = 14.0; CI 95% = 7.9–24.9) times higher prevalence of hair-Hg concentration, respectively, compared with Paapiu. Considering seasonal variation of Hg-exposure, the lowest concentrations were observed during the wet season (June–September) and the highest in the dry season (December–April). Our study suggests that there is an association between mercury exposure and ASGM geographical locations.

## 1. Introduction

Mercury (Hg) exists naturally and as a man-made contaminant. Due to features that include high potential of toxicity, long-term persistence in the environment, global transport by different pathways, and high concentrations in vulnerable areas of the planet, mercury can be considered as one of the principal current environmental concerns [1]. Thinking carefully about these characteristics, delegates from over 140 countries that form the United Nations, signed an international mercury convention in Minamata, Japan, where in the late 1950s the greatest episodes of mercury poisoning took place.

One of the main objectives of the Minamata Convention on Mercury is to reduce anthropogenic use of this metal in order to protect human and environmental health. Artisanal small-scale gold mining (ASGM) represents one of the largest sources (37%) of global anthropogenic mercury emissions [2]. During ASGM, mercury is used to extract gold from the ore through the formation of an amalgam; it is then heated to purify the gold. Consequently, mercury is released into the atmosphere and aquatic ecosystems. The increasing price of gold is the driving force for ASGM in developing countries, such as in South America, Asia, and Africa.

Methylmercury is recognized as one of the most toxic forms of mercury due to its capacity to cross the placenta and blood-brain barrier [1]. If mercury reaches high levels in maternal and fetal circulation, it has the potential to cause irreversible damage in child development, including a reduction of intellectual and motor capacity [2]. Since mercury exposure presents itself with other systemic toxicological effects, over 250 symptoms can complicate accurate diagnosis [3]. However, the main effects concentrate in the nervous, digestive, renal, and cardiovascular systems. In the central nervous system, the effects include depression, paranoia, extreme irritability, hallucinations, memory loss, tremors of the hands, head, lips, and tongue, as well as blindness, retinopathy, optic neuropathy, hearing loss, and a reduced sense of smell [4]. The main symptoms in the digestive system include abdominal pain, indigestion, inflammatory bowel disease, ulcers, and bloody diarrhea [5]. Moreover, mercury can cause kidney damage, including acute tubular necrosis, glomerulonephritis, chronic renal disease, renal cancer, and nephrotic syndrome [6,7,8]. Additionally, mercury accumulation in the heart is associated with cardiomyopathy [9,10].

Transformation of mercury to methylmercury is mediated naturally by microorganisms in aquatic ecosystems. Due to its bioaccumulation and biomagnification properties, methylmercury reaches high levels in top predators of the food chain, such as fish and humans. Therefore, there is a predictable increase in the health risk for populations with fish-based diets, especially in places where mercury is extensively used in ASGM activities [4].

In the Brazilian Amazon, mercury contamination due to ASGM has been reported since the early 1980s. High levels of mercury have been shown by several authors, mainly in riparian populations in that region [11,12,13,14,15,16,17,18]. Despite being recognized as particularly susceptible populations, there are few studies of Hg exposure in indigenous Amazonian communities.

In this context, there are only two studies that look into mercury exposure in the state of Roraima, in the Western Amazon where most of Yanomamis villages are settled [19,20]. ASGM has threatened this traditional population for at least three decades. Besides risk of environmental contamination, deforestation, and loss of natural resources, other damages include social conflicts, diseases and even deaths. Although some actions were taken against ASGM at the end of 1990s, illegal gold extraction has not ceased in that area. Consequently, mercury exposure remains a serious and imminent risk for that population.

Taking into account these potential and real threats, Davi Kopenawa Yanomami, chairman of the Hutukara Yanomami Association (HYA), requested an investigation into mercury exposure in some specific areas of the Yanomami reserve, through a letter addressed to one of the authors (Paulo C. Basta). From this request, our team developed a study that aimed to assess mercury exposure in the Yanomami reserve, as well as if the level of contamination was related to the geographical location of ASGM in the designated areas.

## 2. Materials and Methods

### 2.1. Study Area and Population

**Figure 1:**
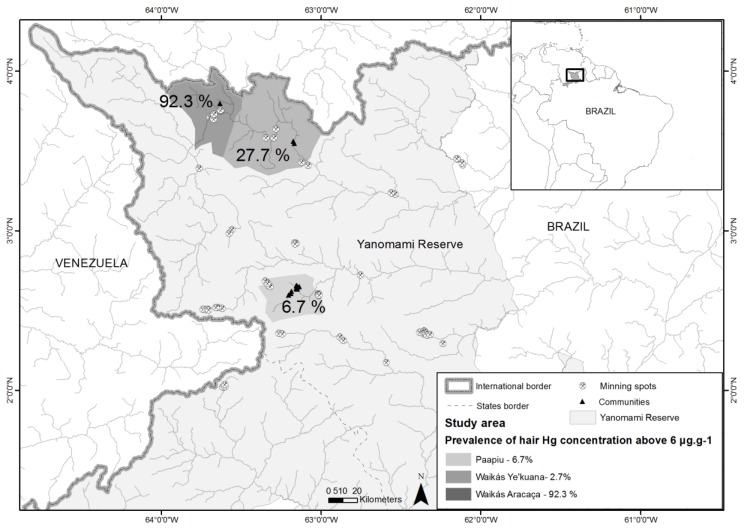
Study area and Prevalence of mercury concentration, considering levels above 6.0 μg·g^−1^, according to villages, Yanomami Reserve, Roraima, Amazon, Brazil, 2014.

The Yanomami are indigenous groups considered to be hunter-gatherers and farmers of traditional slash-fallow (coivara) systems, residing in the Amazon tropical forest. They occupy a territory that extends from the Massif of the Guianas, on both sides of the frontier between Brazil (Upper Branco Basins River, and left bank of the Negro River) and Venezuela (Basins of the Upper Orinoco and Cassiquiare Rivers), in an area that totals 192,000 km^2^. This study was carried out on the Brazilian side, in the Yanomami Indigenous Reserve, located in the Northwest part of the Amazon region (Figure 1).

In Brazil, healthcare for the Yanomami is governed by the Special Sanitary District Yanomami (DSEI-Y, acronym in Portuguese), which is linked to the Special Indigenous Health Secretariat of the Health Ministry. The DSEI-Y is subdivided into 37 Base Stations (considered as Basic Health Units), which provide care to approximately 22,000 indigenous people, including the Yanomami and Ye’kuana ethnic groups, living in 258 villages in the states of Amazonas and Roraima, North region of the country [21,22]. The majority of the villages are located in remote areas, accessible only by air travel or boat. Due to the lack of roads or highways, the seasonal character of navigation and the high costs of air transport, access to health services is extremely limited in these regions.

### 2.2. Study Design

A cross-sectional study was carried out in November and December 2014, in 15 villages attended by the Paapiu Base Station, and four other villages served by the Waikás Base Station. As reported in the introduction, these locations were indicated by the HYA. We obtained demographic details in the selected communities and invited children up to five years old and women of any age, whom were present during the fieldwork to participate. A small number of men who reported working in the ASGM activities were also included. No probabilistic sampling methods were used to select participants because of the selection process.

Paapiu is located on the banks of Mucajai river and is solely populated by Yanomami. In that region intense gold extraction activity was reported during the late 1980s [19]. However, today this activity has been disappearing in this specific area. Waikás is located on the banks of the Uraricoera river, where there are three villages populated by members of the Ye’kuana ethnic group; and one village called Aracaca, located 35 km upriver the Ye’kuanas, where only indigenous from the Yanomami ethnicity live. At the time of the fieldwork, ASGM activity was reported in the Waikás region.

### 2.3. Sample Collection and Variables

The collection of hair samples was carried out by multidisciplinary and multi institutional research groups. Before starting the sample program, several meetings between indigenous communities, their leaders, and the research groups took place at local level.

We included 79 children up to 5 years old; 50 individuals between 6 and 11 years old; and 103 adults (above 12 years old).

For the children and other participants under 18 years old, authorization of the parents and permission was sought. Native interpreters and/or bilingual community leaders, speakers of Portuguese and Yanomami/Ye’kuana accompanied our team during all the fieldwork. The interviews were performed directly with the participants. When children were interviewed, their mothers and/or guardians were invited to answer the questions. The following variables were recorded: date of birth; sex; date of the interview; and home village, in addition to hair samples.

For all the individuals included in the study, hair samples were collected from the occipital area, close to the scalp with stainless steel scissors, which were bundled together with cotton thread and then placed in properly identified paper envelopes.

### 2.4. Total-Hg Determination

Approximately 0.1 g of hair was weighed, dissolved with 1 mL of purified HNO_3_ at room temperature for 12 h, and then heated at 80 °C for 1 h. The next stage involved adding 0.4 mL of H_2_O_2_ to the sample before heating again (80 °C) for 30 min [23]. In addition, there was a sub-sample selected of long hair samples of at least 12 cm, in which Hg analysis in sequential hair segments of 2 cm was performed. This sequential hair analysis was performed in order to investigate changes of Hg concentrations over time in the same individual.

For quality assurance and control, a strict blank control and calibration curve were performed every day. For accuracy, the Certified Reference Material of Human Hair (CRM-13) was analyzed and its recovery rate was above 90%.

All the samples were taken to the laboratory of the Chemistry Department of the Pontificia Universidade Catolica in Rio de Janeiro for analysis performed by Inductive Couple Plasma Mass Spectrometry technique in an ICP-MS 7500 CX (Agilent Technologies, Hanover, Germany).

We used the level above 6.0 μg·g^−1^ as an indicator of health risk, regarding previous studies carried out in Amazon region [15,24,25,26,27,28].

### 2.5. Statistical Analysis

Descriptive analysis was performed on the demographic data, including age groups (up to 5 years old, 6 to 11 years old and greater than 12 years old), sex and place of residence. A chi-square test was used to compare the possible dependency of the prevalence of hair-Hg levels above 6 μg·g^−1^ among various distinct villages. Since Hg-levels presented as a non-normal distribution, the Kruskal-Wallis test was performed to evaluate the differences among the villages.

In order to estimate the prevalence of contamination, we considered the proportion of people who showed levels of mercury above 6.0 μg·g^−1^ out of the population sampled in the study region. The prevalence was presented for three regions (Paapiu, Waikás Ye’kuana, and Waikás Aracaçá).

The Prevalence Ratio (PR) was estimated to provide evidence of association between geographical location of ASGM and human contamination. Simple Poisson regression with a heteroscedasticity-consistent covariance matrix estimator (type HC2) was used [29]. A significance level of 5% (*p* < 0.05) was considered for all statistical tests. The data was analyzed using the R statistical software, version 3.1.1 (R Foundation for Statistical Computing, Vienna, Austria).

### 2.6. Ethical Aspects

This study was performed in accordance with the Declaration of Helsinki. According to the Resolution of the Brazilian National Health Council, which regulates studies involving indigenous populations, written informed consent was read and explained to volunteers and leaders of the community before beginning fieldwork. Due to the high illiteracy rate, oral consent was often obtained with the community leaders as witnesses, and fingerprints were registered by the participants or by parents of children under age.

All data was collected and analyzed anonymously, with written informed consent stored in the office of the principal investigator. The study protocol was approved by the National Commission for Ethics in Research of the National Health Council and the Research Ethics Committee of the National School of Public Health, number 25650713.2.0000.5240.

At the end of the study, individual results with an interpretation of the main results were presented to the communities, in an appropriate language, with support of the local indigenous leaders. A technical report was handed to the Brazilian authorities and the United Nation Special Rapporteur on the rights of indigenous peoples.

## 3. Results

### 3.1. Description of The Studied Population

There were 19 indigenous villages visited, 15 in the Paapiu region, and 4 in the Waikás region. In total, 239 hair samples were analyzed. Samples were obtained from 179 of 360 living in Pappiu, and 60 of 145 living in Waikás, representing 48% and 42% of the entire village population, respectively. Because the Yanomami are hunter-gatherers, a part of the group was not available during the fieldwork.

Considering the age groups of the sampled population, 38% in Paapiu and 35% in Waikas were children up to 5 years old, of which 55% and 76% were female, respectively. In Paapiu, 11.2% and in Waikás, 8.3% were female between 6 and 11 years of age. The group including subjects older than 12 years represented 50.8% in Paapiu and 56.7% in Waikás, of which 76% and 79% were female, respectively. There were no significant differences between the studied regions considering sex and age groups.

### 3.2. Mercury Concentrations in Hair

In the Paapiu region, the population from the 15 villages ranged from 7 to 44 individuals. Due to this variability in population size, an inter-village comparison was not performed. The mercury concentration from each individual ranged from 0.4 to 8.6 μg·g^−1^ and the median value from the 15 villages was 3.2 μg·g^−1^.

**Figure 2:**
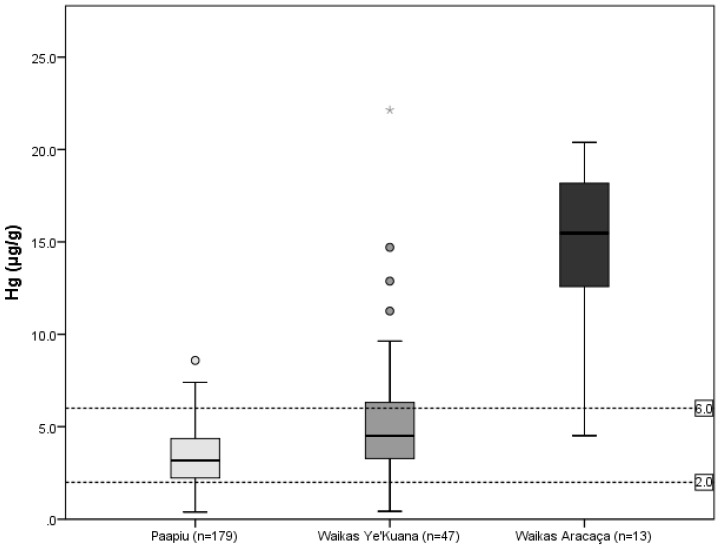
Distribution of total mercury concentration (μg·g^−1^), according to villages: Yanomami reserve, Roraima, Amazon, Brazil, 2014. A significant difference was observed among the three groups (K-W test; *p*-value < 0.001).

In the 3 villages from the Waikás region, where only Ye’kuana indigenous peoples live, individual hair-Hg concentrations ranged from 0.4 to 22.1 μg·g^−1^, with a median of 4.5 μg·g^−1^ for the entire group. No significant differences were observed between these three villages. A small group of Yanomami live in the Aracaça village, upstream on the Uraricoera River, located around 35 km from the Waikas villages, 13 of 29 individuals were sampled. From the 13 of 29 individuals sampled in this village, we found higher concentrations of mercury. The values ranged from 4.6 to 20.4 μg·g^−1^, with a median of 15.5 μg·g^−1^. There was a significant difference among the groups of Paapiu, Waikás Ye’kuana, and Waikás Aracaça (Figure 2).

The prevalence of hair-Hg concentration above 6 μg·g^−1^ was 6.7%, 27.7% and 92.3% for Paapiu, Waikás Ye’kuana, and Waikás Aracaca villages, respectively. The simple Poisson regression analyzed with a heteroscedasticity-consistent covariance matrix estimator showed that the indigenous populations living in Waikas Ye’kuana and Waikas Aracaca villages presented with 4.4 (PR = 4.4; Confidence Interval (CI) 95% = 2.2 to 9.0) and 14.0 (PR = 14.0; CI95% = 7.9 to 24.9) times higher prevalence of hair-Hg concentration above 6 μg·g^−1^ (Table 1) respectively, compared with Paapiu.

### 3.3. Mercury Concentration According to Age Group and Gender

For the entire sampled individuals (*n* = 239), a low significant correlation (Spearman test, R: 0.155, *p* = 0.01) was observed between mercury concentration and age. However, when the three groups (Paapiu, Waikás Ye’kuana, and Waikáas Aracaçá) were analyzed separately, there was no significant correlation between age and Hg concentration. Although there seemed to be a positive and strong association between Hg-levels and age in Aracaça, it did not reach statistical significance. We believe that the lack of statistical significance may be due to the small size of the group (Paapiu R: 0.106; *p*-value = 0.15; Waikás Ye’kuana R: 0.239; *p*-value = 0.06 and Waikás Aracaça R: 0.446, *p*-value = 0.128).

**Figure 3:**
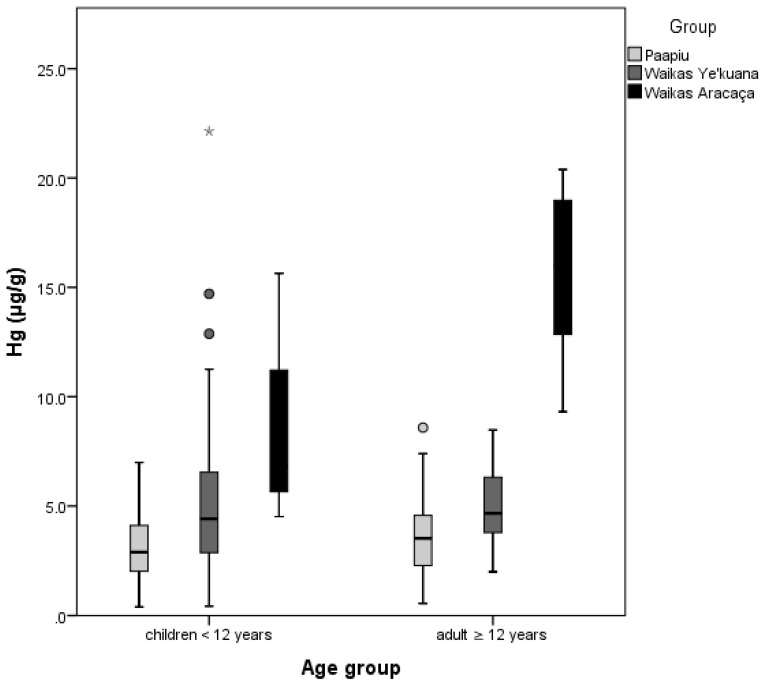
Distribution of total mercury concentrations (µg·g^−1^), according to age groups and villages, Yanomami reserve, Roraima, Amazon, Brazil, 2014.

Taking into account the two age groups (children <12 years and adult ≥12 years), there was no difference in Hg concentrations between children and adults. The difference of Hg concentrations between the three sites was consistent in both age categories. Significantly, higher values were observed in Waikás Aracaça region in comparison with Waikás Ye’kuana and Paapiu (Figure 3).

For the particular case of the Waikás Aracaça village, we would like to highlight some specific findings. We collected hair samples from three girls: one was younger than a year old and two were between one and three years old (0.3, 1.6 and 2.4 years). The mercury concentrations in their hair samples were 4.5, 6.8 and 15.3 μg·g^−1^, respectively. The median value for these children was 9.0 μg·g^−1^. In addition, the median mercury concentration in the adult group reached 16.0 μg·g^−1^, higher than the observed value in the other two studied regions. The adult group included four females (median value 12.7 μg·g^−1^) and six males (median value 17.3 μg·g^−1^). Furthermore, during the 3 h boat ride from Waikás Ye’kuana to Waikás Aracaça villages, our team observed several ASGM camps and boats along the riverbank. It is important to note that the Waikás Ye’kuana community did not report practicing ASGM activities.

As women and children were the focus of the study, there were no analyses in adults related to the association between Hg concentrations and sex. However, both sexes were well represented in children up to 5 years old, and there was no significant difference considering mercury levels between boys and girls. In the adult group, 19 male individuals in Paapiu and 6 in Aracaça were sampled. For the male adults from Paapiu, the median was 3.5 μg·g^−1^, ranging from 1.5 to 8.6 μg·g^−1^. In Waikás Aracaca, the median was considerably higher (17.3 μg·g^−1^), ranging from 14.3 to 20.5 μg·g^−1^.

### 3.4. Seasonal Variation of Hg Concentration in Ye’kuana Women Hair

In order to investigate seasonal variation of mercury concentration, we collected samples of long hair, at least 12 cm, from 8 women from the Waikás Ye’kuana village.

Unfortunately, women from the Paapiu and Waikás Aracaça villages did not have long enough hair to perform this analysis.

**Figure 4:**
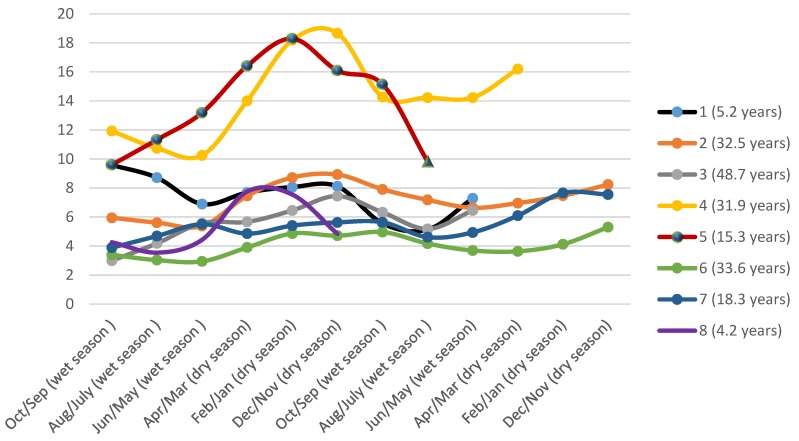
Sequential Hg concentrations (μg·g^−1^), 2 cm of hair segments from each individual, of 8 women from Waikás-Ye’kuana village, Yanomami Reserve, Roraima, Amazon, Brazil, 2014.

Although this subgroup was too small to conduct a more complex statistical analysis, a simple line graph of the results suggests that there might be a seasonal variation in mercury concentrations. Figure 4 shows a rising tendency of Hg in hair in the period between December to April 2014, corresponding to the dry season in the Western Amazon. In addition, lower concentrations were observed during the wet season (between June to September). In the longer hair samples, it was also possible to measure mercury concentrations corresponding to a dry period in 2013, this presented with a similar fluctuation (higher during dry season and lower in wet season) (Figure 4).

## 4. Discussion

Mercury concentrations in the Yanomami reserve, within the Western Amazon, showed substantial differences among the investigated villages. In the Waikás Aracaça region, where current ASGM activities were reported, we observed the highest Hg concentrations in hair (median of 15.5 μg·g^−1^). Furthermore, almost all sampled community members presented hair-Hg levels above 6 μg·g^−1^ (prevalence = 92.3%). Conversely, in the Paapiu region, where ASGM activities took place in 1980s, we observed the lowest concentrations (median of 3.2 μg·g^−1^; prevalence above 6 μg·g^−1^ = 6.7%).

Other studies that focused on exposure to mercury within indigenous populations from the Amazon basin, such as Galabi-Awala from French Guiana [23] and Munduruku from Para State [24], found hair-Hg levels similar to those reported among Paapiu and Ye’Kuana, where the average did not surpass 6 μg·g^−1^.

However, mercury concentration levels above 6 μg·g^−1^ have been reported in other groups with proximity to ASGM activity, such as the Apiaca, Kayapó, Wayani, Munduruku, Wari, Wayan, Kayabi, and Puleowine indigenous groups [26,27,30,31,32,33]. This is in accordance with our results in the Aracaça region that also lies in proximity to ASGM activity.

Considering seasonal variation of Hg-exposure through sequential segmentation of hair strains of Ye’kuana women, our findings showed that the lowest concentrations were observed during the wet season (period between June and September) and the highest in the dry season (between December and April). This might reflect more frequent fish consumption during the dry season, a trend that has also been reported for the riverside populations in the Brazilian Amazon. These populations commonly consume fish during the whole year [34]. However, there is no similar practice within the indigenous populations from the Amazon basin.

One hypothesis for the rise in mercury exposure during the dry season is that the Hg levels are dependent on the quantity of fish consumed, which seems to be higher during the dry season, when fishing practices are easier and more frequent. Moreover, the variation in hair-Hg levels is also attributed to the type of fish consumed, since carnivorous fish have higher levels of Hg than herbivorous ones. According to Sing et al. [20], a high percentage of herbivorous fish are consumed during the wet season and hunting is more often practiced at this time among the Yanomami people. When the traditional populations are exposed to mercury there could be potential neurotoxic, and immuno-toxic effects, adversely affecting the cognitive and motor development of children. Despite the critical effects to human health, there have been no studies evaluating these clinical symptoms within the indigenous population from the Brazilian Amazon.

Another factor that could further exacerbate mercury exposure is the limited access to basic health services as well as the continuous exposure to illnesses that are endemic in that region, such as malaria, tuberculosis, acute respiratory conditions, and onchocerciasis [35,36]. These are also associated with high levels of malnutrition [21,37]. With these conditions acting in synergy, this population becomes more vulnerable to a broad variety of health problems, including mercury contamination and its consequences.

Although fish consumption plays an important role in Hg exposure, our study observed that the communities with direct involvement in gold mining activities presented with the higher concentrations of hair-Hg.

As indigenous populations are highly dependent on their natural environment, it is important to reduce any factor that could jeopardize this environment. When referring to ASGM, mercury contamination and deforestation are among the factors that negatively affect the Amazonian ecosystem and the populations (human and non-human) that inhabit this area.

One of the main limitations of this study was the lack of a systematic diet assessment. We recognize the importance of the diet in understanding mercury contamination patterns, however we would like to highlight the difficulty of this approach in indigenous populations. First, among many ethnicities it is not possible to communicate with the individual without a translator, which was the case with the Yanomami, where we needed to collect data accompanied by at least one speaker of Portuguese and Yanomami. Second, in general, for indigenous populations from the Amazon basin, the availability of food (game meat, roots, and fish, among others) varies according to the period of the year (seasonality). Sometimes, in the wet season, the whole community eats the same food for extended periods of time. Third, time reference and feeding habits of these communities are different from Western patterns. Therefore, methods commonly used for diet surveys in non-indigenous populations, such as a 7 day recall, could not be used. Thus, we believe that a more direct method, such as observation over a specific period should be considered.

Another limitation was the lack of clinical information about the studied group. We had access only to birth date from the health agent’s registration books. At the end of the study, both individual, and overall results were presented to the communities, and a technical report was handed to the Brazilian authorities. Unfortunately, deeper clinical evaluation in the subjects who showed levels of mercury above 6 μg·g^−1^ was not possible due to lack of time and resources.

## 5. Conclusions

Our study shows how levels of hair-Hg above those that are considered as reference values to the Amazon are still prevalent in indigenous populations residing in protected areas of the Brazilian Amazon. Although high levels of mercury in this region have been reported previously, our study was designed to give practical answers to the communities. We worked in close collaboration with leadership communities and a survey was carried out following a request from the indigenous association. Moreover, the results of hair-Hg levels were presented and explained to all the communities involved in the study. These results were used as a political tool in order to demand interventions from national and international authorities. For the first time, indigenous communities from the Brazilian Amazon have scientific evidence that ASGM causes a significant Hg exposure in indigenous peoples.

The neglected health situation observed in the Yanomami reserve during the fieldwork points out the complexity of the mercury exposure risk in the indigenous communities in the Amazon.

The indiscriminate use of mercury in gold mining extraction, together with the lack of law enforcement by local and federal government ensuring there is no ASGM invasion in protected indigenous land, compromise both the way of life and the sustainable development of these populations. This is further compounded by the deforestation, the cultural and social interference in the traditional communities, the limited access to basic health services and the permanent exposure to infectious diseases.

In conclusion, we believe that to ensure the preservation, food safety, and health of the indigenous populations in Brazil, it is vital that the nation achieves the Sustainable Development Goals proposed by the United Nations. The Brazilian government must develop policies and strategies in order to ensure food quantity and quality, access to clean water, human rights, land and health services, and preservation of traditional cultures in all national territory.

## Figures and Tables

**Table 1:** Prevalence of mercury concentration, considering levels above 6.0 μg·g^−1^, and Prevalence Ratio to illustrate a possible association between ASGM geographical location and mercury exposure, Yanomami reserve, Roraima, Amazon, Brazil, 2014.

Villages	≥6 μg·g^−1^	<6 μg·g^−1^	Total Sampled	Entire Population	Prevalence	PR	CI 95%
Waikás Aracaça	12	1	13	29	92.3	14.0	7.9–24.9
Waikás Yekuana	13	34	47	116	27.7	4.4	2.2–9.0
Paapiu	12	167	179	360	6.7		
Total	37	202	239	505	15.5		

PR: Prevalence-Ratio. CI: Confidence Interval.
